# mRNA modifications: Dynamic regulators of gene expression?

**DOI:** 10.1080/15476286.2016.1203504

**Published:** 2016-06-28

**Authors:** Thomas Philipp Hoernes, Alexander Hüttenhofer, Matthias David Erlacher

**Affiliations:** Division of Genomics and RNomics, Biocenter, Medical University of Innsbruck, Innsbruck, Austria

**Keywords:** Gene expression, mRNA modifications, ribosome, translation regulation

## Abstract

The expression of a gene is a tightly regulated process and is exerted by a myriad of different mechanisms. Recently, RNA modifications located in coding sequences of mRNAs, have been identified as potential regulators of gene expression. *N*^6^-methyladenosine (m^6^A), 5-methylcytosine (m^5^C), pseudouridine (Ψ) and *N*^1^-methyladenosine (m^1^A) have been found within open reading frames of mRNAs. The presence of these mRNA modifications has been implicated to modulate the fate of an mRNA, ranging from maturation to its translation and even degradation. However, many aspects concerning the biological functions of mRNA modifications remain elusive. Recently, systematic *in vitro* studies allowed a first glimpse of the direct interplay of mRNA modifications and the efficiency and fidelity of ribosomal translation. It thereby became evident that the effects of mRNA modifications were, astonishingly versatile, depending on the type, position or sequence context. The incorporation of a single modification could either prematurely terminate protein synthesis, reduce the peptide yield or alter the amino acid sequence identity. These results implicate that mRNA modifications are a powerful mechanism to post-transcriptionally regulate gene expression.

## RNAs involved in the regulation of gene expression

Regulation of gene expression is a complex multistep process. The synthesis of a functional protein is subject to several layers of regulation, starting from the synthesis of various transcription factors up to the correct assembly of the nascent protein by chaperones. The most direct mechanism of regulating protein synthesis is the modulation of the amounts of messenger RNAs (mRNAs) within a cell. However, a direct correlation between the amounts of an mRNA and its corresponding protein is not always observed.^1,2^ Hence, protein synthesis is the target of various highly sophisticated regulatory mechanisms, of which more and more have been identified in the last decade.^3-9^

Generally, protein synthesis can be divided into 4 stages: initiation, elongation, termination, and ribosome recycling. Traditionally, the initiation step is viewed as one key feature of regulation and numerous factors and mechanisms have been described that lead either to a global or an mRNA-specific initiation control.^3,4,10^ Not only altering amounts and activities of initiation factors, but also the presence of regulatory sequences or structural motifs in the 5′ or 3′ untranslated regions (UTRs) of mRNAs, are now well-understood factors for regulating initiation of protein synthesis.^3,4,11,12^

Equally important for regulation of translation are RNA binding proteins (RBPs) and non-coding RNAs (ncRNAs). As soon as ncRNAs, such as miRNAs and siRNAs, had been identified, it became evident that these small ncRNA species are directly involved in modulating gene expression.^13,14^ Although siRNA and miRNA differ in their origin and function, both guide the RNA Induced Silencing Complex (RISC) to their target mRNAs and thus induce cleavage or degradation of mRNAs, respectively. Thereby, miRNAs were reported to modulate translation initiation as well as elongation, thus rendering small ncRNAs as versatile molecules for modulating gene expression.^15^

Whereas RBPs and ncRNAs bind to mRNAs, a recently discovered class of regulatory RNAs directly binds to the ribosome, thereby affecting protein synthesis. These ribosomal associated ncRNAs (rancRNAs) interfere with protein synthesis in a stress-dependent manner.^16-18^ Thereby, RNA fragments derived from mRNAs or transfer RNAs (tRNAs) have been identified to bind to the ribosome and globally inhibit translation. For example, 5′-tRNA fragments, identified in *Haloferax volcanii*, downregulate protein synthesis globally by binding to the small ribosomal subunit, thereby competing with binding of mRNAs. Subsequently, additional tRNA fragments have been identified to interfere with translation,^19-21^ but their biological roles and mechanisms of action are still not completely understood.^22^

Since also full-length tRNAs interact with the ribosome and thus play a central role in translation, it seems inevitable that they would be also involved in modulating translation. Thereby, it is has been reported that the abundance, the availability of tRNAs and the codon usage within mRNAs strongly influences the speed and efficiency of translation (^23,24^ and reviewed in^25,26^). Recently, tRNA modifications were also revealed to be linked to translation efficiency and decoding fidelity.^26-28^ In addition, modifications of tRNAs have been found to be involved in fine tuning of stress-related genes by driving codon biased translation.^7,29^

In addition, mRNAs are not just mere templates for translation, but harbor essential regulatory elements. As mentioned above, specific regions within UTRs of mRNAs might be involved in regulation of translation.^3,4,11,12^ These regulatory elements, such as structural RNA motifs or binding sites for proteins or for regulatory RNAs, are found primarily in 5′ and 3′ UTRs of mRNAs. However, also ORFs are able to influence the efficiency and speed of translation.^30-32^ Thereby, specific codons and sequence elements cause ribosomal stalling and consequently the folding and activity of the produced proteins might be affected.^24,33-36^ Codon-optimized sequences might result in higher product yields, but also lead to lower enzyme activities.^34^

Recently, a mechanism in regulating ribosomal translation has been identified: thereby, modifications of RNA nucleotides within coding sequences of mRNAs were reported to directly interfere with elongation and decoding of the ribosomal translation machinery.^37-39^ Herein, we summarize current findings of co- and post-transcriptional mRNA modifications affecting translation and anticipate what lessons these modifications might teach us (i.e. what biological roles they might exert in different organisms).

## Old RNA modifications - new perspectives

Modifications within the ORFs of mRNAs have already been described in the 1970s, demonstrating the occurrence of *N*^6^-methyladenosine (m^6^A)^40-44^ and 5-methylcytosine (m^5^C).^45^ Due to technical limitations, however, the identification of distinct nucleotide modifications was not possible. Thus, neither the detailed localization of modifications within transcripts, nor their biological role has been elucidated. Hence, these early studies were sometimes controversially interpreted in respect to a potential function of mRNA modifications, mainly due to their presumably low abundance and their identification in distinct cell types only.^40,44^ But subsequent to uncovering specific enzymes, capable of converting adenosines (A) to inosines (I) within dsRNA (ADARs),^46-51^ the field of mRNA modifications re-gained attention. This A-to-I editing process has been unveiled as the most prevalent form of mRNA editing, leading to a new and prospering research field.

The interest in mRNA nucleotide modifications once again increased through the invention of RNA mass spectrometry and next generation sequencing (NGS) techniques, which equipped researchers with powerful tools to identify and map modifications within transcripts. Several groups have contributed to our current understanding of when, where, and most importantly how mRNA modifications regulate gene expression. To date, the repertoire of naturally occurring eukaryal mRNA modifications (besides the 5´ cap and inosine) is comprised of m^6^A, pseudoruridine (Ψ), m^5^C and *N*^1^-methyladenosine (m^1^A).^52-59^ Thereby, m^6^A is not only the most abundant, but also the best-characterized internal mRNA modification so far. The dynamic nature of the presence of m^6^A within mRNAs and its involvement in various biological functions is remarkable, ranging from splicing, regulation of translation to mRNA decay.^60-63^ Also Ψ is dynamically deposited in mRNA transcripts, but its function within mRNAs is still elusive.^53-55^

Even less is known about the possible role of m^5^C within mRNAs. m^5^C was reported to be present in human^56^ as well as in archaeal^59^ mRNAs. Due to its reported enrichment within the UTRs of human mRNAs and its location in the vicinity of Argonaute binding sites, m^5^C was suggested to be involved in translation regulation.^56^ The most recent modification, which has joined the mRNA repertoire, is m^1^A.^57,58^ Thereby, m^1^A is predominantly found in structured regions of the 5′ UTR of mRNAs and in the vicinity of canonical and alternative translation initiation sites. Most interestingly, the presence of m^1^A in mRNAs is connected to elevated translation rates.^57^ Modifications of the ribose, such as 2′-O-methylations, have not yet been unambiguously identified within coding sequences of mRNAs. 2′-O-methylations are commonly found in ribosomal RNAs (rRNAs), small nuclear RNAs (snRNAs, reviewed in^64^) or tRNAs (reviewed in^65^) and are also speculated to be introduced into ORFs by small nucleolar RNA-guided complexes.^66,67^

## Translational regulation mediated by mRNA modifications

Until recently, mRNA modifications have only been indirectly linked to ribosomal translation.^61,62,68-71^ An emerging body of evidence implies that the effect of modifications is not only dependent on its type but also on the translation system. Thereby, mRNAs harboring Ψs within coding sequences increased the protein yield in rabbit reticulocyte extracts but not in wheat germ extracts.^70^ However, in bacterial translation systems mRNAs with randomly incorporated Ψs strongly inhibited protein synthesis.^70^ Similar observations have been made with other mRNA modifications as well.^68,70^ These findings were supported by cell culture-based approaches revealing a significant cell type dependency.^57,70,71^

Additionally, the sequence context affects the impact of mRNA modifications. Thereby, protein expression strongly depends on the corresponding mRNA sequence, thus making it even more complicated to univocally draw conclusions about the impact of specific modifications on the translation machinery.^70,71^

The majority of these studies employed randomly modified mRNAs.^68-70^ Alternatively, mRNAs with a complete substitution of the unmodified nucleotide by a modified version were applied.^71^ Thus, in order to obtain a more detailed picture of the direct effect of single mRNA modifications on protein synthesis, a refined systematic approach was applied. Employing a splinted ligation protocol, RNA modifications were site-specifically incorporated into reporter mRNAs.^37^ Thereby, the nucleotide derivatives were positioned at the 1^st^, 2^nd^ or 3^rd^ position of the codon, respectively, and subsequently peptide products of the corresponding mRNA construct were analyzed.

Strikingly, the resulting effects on translation were not only strongly dependent on the type but also on the position of the modifications. Thereby, 2′-O-methylated nucleosides at the 1^st^ codon position only marginally affected translation, however, when placed at the 2^nd^ position they caused an almost complete termination of protein synthesis at the modified nucleotide.^37^ On the contrary, m^6^A revealed the strongest inhibition at the 1^st^ and Ψ at the 3^rd^ codon position.^37,38^ In addition, also the sequence context seemed to exert a significant influence on translation. Whereas the 2′-O-methyl group at the second codon position was independent of the codon and the sequence context,^37^ m^6^A exhibited a strong sequence dependence.^38^

In these studies, not only the efficiency of translation was investigated but also a long-standing question concerning the ability of mRNA modifications to rewire the genetic code was addressed. Thereby, the best-known example of recoding the genetic information at the RNA level is A-to-I editing.^72,73^ Within coding sequences I is read as a G by the translation machinery and therefore can lead to an amino acid change, dependent on its position within a codon.

It has been speculated that Ψ within coding sequences might also possess the potential to rewire the genetic code.^53,74-76^ This speculation was based on observations that Ψ, located within a stop codon caused partial read-through of translation.^75^ However, in coding sequences a recoding event, induced by Ψs could not be detected.^37^ In contrast, m^5^C induced, to some extent, an amino acid substitution.^37^ Thereby, m^5^C placed at the 2^nd^ position of a CCC codon, resulted in a substitution of proline by leucine. Importantly, this partial “recoding” was also strongly dependent on the position of the modification within the codon and might also be influenced by the sequence context, similar to m^6^A.^38^ Thus, additional experiments will be required to identify mechanisms how m^5^C interferes with ribosomal decoding. It further needs to be demonstrated, if this rather weak recoding effect is of biological relevance. Nevertheless, it is remarkable that the decoding process of the bacterial translation machinery is affected by m^5^C in a codon position-dependent manner.

## Potential functions of internal mRNA modifications

Dependent on the modification and the position within a codon, a variety of effects on protein synthesis have been observed. Introducing a 2′-O-methyl group at the 2^nd^ codon position prematurely terminated translation efficiently at the site of modification. This poses the question, as to the function of translation termination at the modification site and the associated peptide fragments. As a very direct consequence, modifications might merely reduce the amounts of a protein produced by the modified mRNA, thereby regulating its expression in analogy to miRNAs, for example. It is thereby interesting to note that in eukarya, mRNAs containing premature stop codons, which would result in such shortened peptides, are immediately degraded by the nonsense mediated decay machinery (NMD, reviewed in^77^).

In bacteria, mRNAs lacking a stop codon due to shortening of the mRNA by degradation or cleavage, are subject to the tmRNA pathway (reviewed in^78^). Thereby, the transfer-messenger RNA (tmRNA), binds to the ribosomal A site, by first structurally mimicking an alanine tRNA, and adding alanine to the peptide chain.^79,80^ Subsequently, the tmRNA acts as an mRNA adding a specific protein sequence, encoded within the tmRNA, to the truncated peptide that is subsequently recognized and degraded by a protease.^81^ Thus, in both eukarya and bacteria, these mechanisms prevent the generation of shortened peptides that might be harmful to cells.

In this context, site-specific incorporation of certain modifications within mRNAs might have a function, in addition to merely reducing protein levels: in particular, they might enable the generation of shortened peptides with novel functions, beneficial for eukarya or bacteria, respectively. Dependent on the level of modification within an mRNA and its position, various amounts of these peptides can be generated and peptides of various sizes, exhibiting different functions, might be produced. The benefit of such a mechanism is that, despite the presence of the modification and its impact on translation, in addition the full-length protein could still be synthesized in sufficient levels, thereby increasing protein diversity.

In fact, there are examples which support such a model: apoliprotein B (apoB) is synthesized in the liver as apoB100, whereas in the small intestine the apoB48 variant is present.^82,83^ Both proteins are produced by the same gene, but through C-to-U editing in the small intestine a UAA codon is generated, resulting in the truncated form of the protein with distinct functions compared with full-length apoB100.^84^

In addition, regulatory mechanisms exist that induce these mRNA modifications only in response to certain environmental cues. Such a dependency has been demonstrated for Ψ, m^6^A and very recently for m^1^A.^52,53,57^ These mechanisms thus might reflect an epigenetic regulation of gene expression on the level of RNA, where signals from the environment of a cell are transferred to RNAs. However, if mRNA modifications in fact represent such a so far unidentified mechanism to generate smaller, but functional, peptides of various length, still the question has to be answered, how the ribosome deals with such a premature termination, in particular as release factors are required to free the premature terminated peptide from the ribosome.

**Figure 1.**
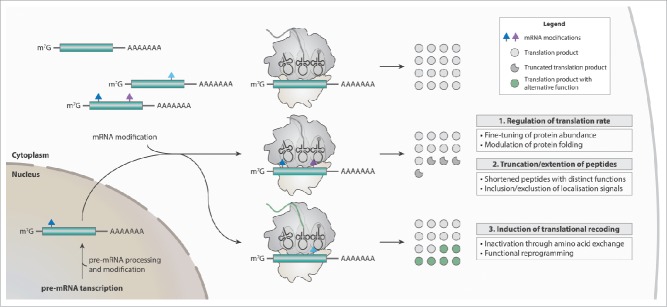
The potential cellular functions mediated by mRNA modifications are versatile. Modifications can be co- or post-transcriptionally incorporated into mRNAs (green) in response to environmental stimuli, such as changes in growth conditions or stress (e.g. starvation, radiation, temperature shifts, nutrition deprivation etc.). Single modifications or combinations thereof within an mRNA might regulate the rate of translation, the actual peptide length, or the identity of a protein. Thus, mRNA modifications represent a highly sophisticated biological mechanism to regulate gene expression.

In summary, the site-specific incorporation of modified RNA nucleotides into coding regions of mRNAs revealed astonishingly versatile effects on protein synthesis depending not only on the type of the RNA modification but also on the codon position (Fig. 1). In addition, various organisms and cell types potentially cope differently with the presence of modifications within mRNAs. Their biological function can range from fine-tuning translational rates to premature termination of protein synthesis. Post-transcriptional mRNA modifications might even possess the potential to expand the diversity of proteins through recoding. Therefore, it is of utmost importance to elucidate all mechanisms behind.

mRNA modifications not only affect translation, but can also act as markers to provide landing platforms for proteins^61,62,85,86^ or stimulate other regulatory processes like mRNA degradation^60^ or localization.^87^ Their role as markers is reminiscent of the regulation of gene expression through epigenetic DNA and histone modifications. In line with that, not single modifications but a combination thereof might collectively mediate biological functions. Such modification patterns could serve as landmarks to stimulate or trigger down-stream effects. However, this is purely speculative and many aspects of mRNA modifications are still far from being completely understood. Elucidating the regulation of mRNA modifications and their cellular functions will open up a completely new way in understanding gene regulation on the level of RNA.
